# *N*-{(*E*)-[4-(Dimethyl­amino)­phen­yl]methyl­idene}-2,3-dimethyl­aniline

**DOI:** 10.1107/S1600536810027832

**Published:** 2010-07-17

**Authors:** Muhammad Sarfraz, Muhammad Ilyas Tariq, M. Nawaz Tahir

**Affiliations:** a Department of Chemistry, University of Sargodha, Sargodha, Pakistan; b Department of Physics, University of Sargodha, Sargodha, Pakistan

## Abstract

There are two independent mol­ecules in the asymmetric unit of the title compound, C_17_H_20_N_2_, in which the dihedral angles between the aromatic rings are 30.34 (11) and 41.44 (8)°. In the crystal, weak C—H⋯π inter­actions may help to establish the packing.

## Related literature

For related structures, see: Hussain *et al.* (2010 ▶); Tahir *et al.* (2010*a* ▶,*b* ▶); Tariq *et al.* (2010 ▶).
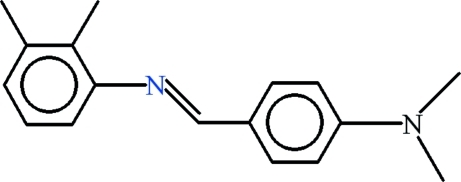


## Experimental

### 

#### Crystal data

C_17_H_20_N_2_
*M* *_r_* = 252.35
Triclinic, 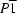

*a* = 7.6556 (8) Å
*b* = 7.7296 (8) Å
*c* = 25.059 (3) Å
α = 93.843 (6)°
β = 95.436 (6)°
γ = 97.431 (5)°
*V* = 1459.1 (3) Å^3^
*Z* = 4
Mo *K*α radiation
μ = 0.07 mm^−1^
*T* = 296 K
0.28 × 0.18 × 0.16 mm

#### Data collection

Bruker Kappa APEXII CCD diffractometer
Absorption correction: multi-scan (*SADABS*; Bruker, 2005 ▶) *T*_min_ = 0.982, *T*_max_ = 0.988
20871 measured reflections
5142 independent reflections
2482 reflections with *I* > 2σ(*I*)
*R*_int_ = 0.075

#### Refinement

*R*[*F* ^2^ > 2σ(*F* ^2^)] = 0.065
*wR*(*F* ^2^) = 0.188
*S* = 1.02
5142 reflections
352 parameters
H-atom parameters constrained
Δρ_max_ = 0.49 e Å^−3^
Δρ_min_ = −0.50 e Å^−3^

### 

Data collection: *APEX2* (Bruker, 2009 ▶); cell refinement: *SAINT* (Bruker, 2009 ▶); data reduction: *SAINT*; program(s) used to solve structure: *SHELXS97* (Sheldrick, 2008 ▶); program(s) used to refine structure: *SHELXL97* (Sheldrick, 2008 ▶); molecular graphics: *ORTEP-3* (Farrugia, 1997 ▶) and *PLATON* (Spek, 2009 ▶); software used to prepare material for publication: *WinGX* (Farrugia, 1999 ▶) and *PLATON*.

## Supplementary Material

Crystal structure: contains datablocks global, I. DOI: 10.1107/S1600536810027832/hb5549sup1.cif

Structure factors: contains datablocks I. DOI: 10.1107/S1600536810027832/hb5549Isup2.hkl

Additional supplementary materials:  crystallographic information; 3D view; checkCIF report

## Figures and Tables

**Table 1:** Hydrogen-bond geometry (Å, °) *Cg*1, *Cg*2 and *Cg*4 are the centroids of the C1–C6, C10–C15 and C27–C32 benzene rings, respectively.

*D*—H⋯*A*	*D*—H	H⋯*A*	*D*⋯*A*	*D*—H⋯*A*
C7—H7*A*⋯*Cg*1^i^	0.96	2.90	3.756 (3)	149
C16—H16*C*⋯*Cg*4^ii^	0.96	2.69	3.434 (4)	135
C32—H32⋯*Cg*2^iii^	0.93	2.88	3.698 (3)	148

Symmetry codes: (i) 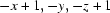; (ii) 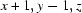; (iii) 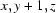.

**Fig. 1.**
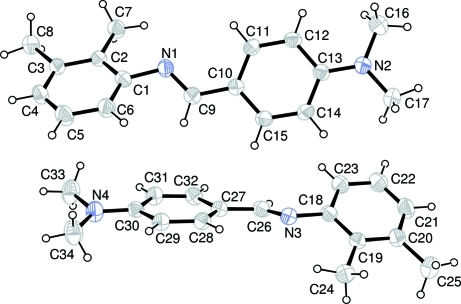
View of (I) with displacement ellipsoids drawn at the 30% probability level.

## supplementary crystallographic information

### Comment

The title compound (I, Fig. 1) is being reported in continuation to synthesize various Schiff bases (Hussain *et al.*, 2010; Tahir *et al.*, 2010*a*; Tahir *et al.*, 2010*b*; Tariq *et al.*, 2010) of 2,3-dimethylaniline.

The crystal structures of (II) i.e, 2,3-dimethyl-*N*-[(*E*)-4-nitrobenzylidene]aniline (Tariq *et al.*, 2010), (III) *N*-[(*E*)-4-chlorobenzylidene]-2,3-dimethylaniline (Tahir *et al.*, 2010*a*), (IV) (*E*)-2,3-dimethyl-*N*-(2-nitrobenzylidene)aniline (Tahir *et al.*, 2010*b*) and (V) 2,3-dimethyl-*N*-[(*E*)-2,4,5-trimethoxybenzylidene]aniline (Hussain *et al.*, 2010) have been published which contain 2,3-dimethylaniline moiety. The title compound differs from these due to substitutions at the benzene ring of the aldehyde moiety.

The title compound consists of two molecules in the crystallographic asymmetric unit which differ from each other geometrically. In one molecule, the group A (C1—C8) of 2,3-dimethylaniline and the group B (C9—C15) of *N*,*N*^'^-dimethylbenzaldehyde are planar with r. m. s deviation of 0.009 and 0.017 Å, respectively. The *N*,*N*^'^-dimethyl group C (C16/N2/C17) is of course planar. The dihedral angle between A/B, A/C and B/C is 41.08 (07)°, 38.24 (19)° and 5.17 (37)°, respectively. In second molecule, the group D (C18—C25) and group E (C26—C32) are also planar with r. m. s deviation of 0.010 and 0.018 Å, respectively. The dihedral angle between D/E is 30.44 (09)°. The *N*,*N*^'^-dimethyl group F (C33/N4/C34) of this molecule makes dihedral angle of 18.40 (17)° with group D, whereas it is oriented at 12.14 (37)° with group E. In first molecule, the N-atom of 2,3-dimethylaniline is at a distance of 0.1105 (37) Å from the mean square plane of group A. In comparison to this, in second molecule, the N3 is at -0.0152 (41) Å from group D and shows that it is in plane to D. The title compound is stabilized due to weak C—H···N and C—H···π interactions (Table 1).

### Experimental

Equimolar quantities of 2,3-dimethylaniline and 4-(dimethylamino)benzaldehyde were refluxed in methanol for 2.5 h. The yellow solution obtained was kept at room temperature to affoard colorless prisms of (I) in 12 h.

### Refinement

All H-atoms were positioned geometrically (C–H = 0.93, 0.96 Å) and refined as riding with *U*_iso_(H) = *xU*_eq_(C), where *x* = 1.2 for aryl and *x* = 1.5 for methyl H-atoms.

### Crystal data

**Table:**

C_17_H_20_N_2_	*Z* = 4
*M**_r_* = 252.35	*F*(000) = 544
Triclinic, *P*1	*D*_x_ = 1.149 Mg m^−^^3^
Hall symbol: -P 1	Mo *K*α radiation, λ = 0.71073 Å
*a* = 7.6556 (8) Å	Cell parameters from 2482 reflections
*b* = 7.7296 (8) Å	θ = 2.5–25.1°
*c* = 25.059 (3) Å	µ = 0.07 mm^−^^1^
α = 93.843 (6)°	*T* = 296 K
β = 95.436 (6)°	Prism, colorless
γ = 97.431 (5)°	0.28 × 0.18 × 0.16 mm
*V* = 1459.1 (3) Å^3^	

### Data collection

**Table:**

Bruker Kappa APEXII CCD diffractometer	5142 independent reflections
Radiation source: fine-focus sealed tube	2482 reflections with *I* > 2σ(*I*)
graphite	*R*_int_ = 0.075
Detector resolution: 8.20 pixels mm^-1^	θ_max_ = 25.1°, θ_min_ = 2.5°
ω scans	*h* = −9→9
Absorption correction: multi-scan (*SADABS*; Bruker, 2005)	*k* = −9→9
*T*_min_ = 0.982, *T*_max_ = 0.988	*l* = −29→29
20871 measured reflections	

### Refinement

**Table:**

Refinement on *F*^2^	Secondary atom site location: difference Fourier map
Least-squares matrix: full	Hydrogen site location: inferred from neighbouring sites
*R*[*F*^2^ > 2σ(*F*^2^)] = 0.065	H-atom parameters constrained
*wR*(*F*^2^) = 0.188	*w* = 1/[σ^2^(*F*_o_^2^) + (0.0834*P*)^2^] where *P* = (*F*_o_^2^ + 2*F*_c_^2^)/3
*S* = 1.02	(Δ/σ)_max_ < 0.001
5142 reflections	Δρ_max_ = 0.49 e Å^−^^3^
352 parameters	Δρ_min_ = −0.50 e Å^−^^3^
0 restraints	Extinction correction: *SHELXL97* (Sheldrick, 2008), Fc^*^=kFc[1+0.001xFc^2^λ^3^/sin(2θ)]^-1/4^
Primary atom site location: structure-invariant direct methods	Extinction coefficient: 0.079 (6)

### Special details

**Table:**

Geometry. Bond distances, angles *etc*. have been calculated using the rounded fractional coordinates. All su's are estimated from the variances of the (full) variance-covariance matrix. The cell e.s.d.'s are taken into account in the estimation of distances, angles and torsion angles
Refinement. Refinement of *F*^2^ against ALL reflections. The weighted *R*-factor *wR* and goodness of fit *S* are based on *F*^2^, conventional *R*-factors *R* are based on *F*, with *F* set to zero for negative *F*^2^. The threshold expression of *F*^2^ > σ(*F*^2^) is used only for calculating *R*-factors(gt) *etc*. and is not relevant to the choice of reflections for refinement. *R*-factors based on *F*^2^ are statistically about twice as large as those based on *F*, and *R*- factors based on ALL data will be even larger.

### Fractional atomic coordinates and isotropic or equivalent isotropic displacement parameters (Å^2^)

**Table:**

	*x*	*y*	*z*	*U*_iso_*/*U*_eq_	
N1	0.5198 (3)	0.0581 (3)	0.37159 (10)	0.0537 (9)	
N2	0.9529 (3)	−0.1392 (4)	0.17264 (10)	0.0721 (11)	
C1	0.3947 (4)	0.1128 (4)	0.40574 (12)	0.0515 (11)	
C2	0.4576 (3)	0.1876 (3)	0.45733 (11)	0.0494 (11)	
C3	0.3346 (4)	0.2293 (4)	0.49253 (12)	0.0543 (11)	
C4	0.1557 (4)	0.1959 (4)	0.47532 (14)	0.0660 (12)	
C5	0.0951 (4)	0.1219 (4)	0.42452 (14)	0.0735 (15)	
C6	0.2148 (4)	0.0822 (4)	0.38967 (12)	0.0626 (12)	
C7	0.6526 (4)	0.2190 (4)	0.47505 (11)	0.0648 (11)	
C8	0.3963 (4)	0.3060 (4)	0.54930 (12)	0.0712 (12)	
C9	0.4960 (4)	0.0717 (4)	0.32134 (12)	0.0534 (11)	
C10	0.6111 (3)	0.0102 (3)	0.28366 (11)	0.0478 (10)	
C11	0.7562 (4)	−0.0742 (3)	0.29931 (11)	0.0500 (10)	
C12	0.8662 (3)	−0.1263 (4)	0.26331 (11)	0.0517 (11)	
C13	0.8376 (4)	−0.0954 (4)	0.20859 (11)	0.0501 (11)	
C14	0.6900 (4)	−0.0152 (4)	0.19267 (11)	0.0581 (11)	
C15	0.5813 (4)	0.0354 (4)	0.22929 (11)	0.0551 (11)	
C16	1.1113 (4)	−0.2115 (5)	0.18959 (13)	0.0881 (16)	
C17	0.9234 (5)	−0.1121 (5)	0.11657 (13)	0.0966 (18)	
N3	0.4573 (3)	0.3231 (3)	0.13425 (10)	0.0582 (10)	
N4	0.0318 (3)	0.4923 (3)	0.33878 (10)	0.0677 (10)	
C18	0.5873 (4)	0.3266 (4)	0.09694 (12)	0.0552 (11)	
C19	0.5286 (4)	0.2815 (4)	0.04280 (13)	0.0580 (12)	
C20	0.6535 (5)	0.2793 (4)	0.00571 (13)	0.0687 (12)	
C21	0.8323 (5)	0.3209 (4)	0.02383 (15)	0.0770 (16)	
C22	0.8891 (4)	0.3616 (4)	0.07720 (15)	0.0773 (16)	
C23	0.7672 (4)	0.3641 (4)	0.11396 (13)	0.0685 (14)	
C24	0.3328 (4)	0.2381 (5)	0.02473 (12)	0.0775 (14)	
C25	0.5962 (5)	0.2378 (5)	−0.05378 (13)	0.0969 (19)	
C26	0.4823 (4)	0.4361 (4)	0.17434 (12)	0.0580 (11)	
C27	0.3656 (4)	0.4420 (4)	0.21654 (12)	0.0522 (11)	
C28	0.2033 (4)	0.3369 (4)	0.21470 (11)	0.0539 (11)	
C29	0.0937 (4)	0.3511 (4)	0.25459 (11)	0.0541 (11)	
C30	0.1432 (4)	0.4714 (4)	0.29963 (11)	0.0507 (11)	
C31	0.3100 (4)	0.5717 (4)	0.30267 (12)	0.0597 (12)	
C32	0.4155 (4)	0.5572 (4)	0.26191 (12)	0.0581 (11)	
C33	0.0955 (5)	0.5875 (5)	0.38990 (13)	0.0877 (16)	
C34	−0.1493 (4)	0.4123 (5)	0.33187 (13)	0.0852 (16)	
H4	0.07449	0.22425	0.49869	0.0791*	
H5	−0.02589	0.09880	0.41383	0.0880*	
H6	0.17451	0.03442	0.35496	0.0751*	
H7A	0.67625	0.16211	0.50736	0.0974*	
H7B	0.69064	0.34244	0.48182	0.0974*	
H7C	0.71577	0.17235	0.44731	0.0974*	
H8A	0.29609	0.33240	0.56706	0.1070*	
H8B	0.47713	0.41138	0.54830	0.1070*	
H8C	0.45501	0.22305	0.56858	0.1070*	
H9	0.39978	0.12387	0.30800	0.0639*	
H11	0.77847	−0.09560	0.33523	0.0600*	
H12	0.96108	−0.18284	0.27508	0.0620*	
H14	0.66535	0.00399	0.15666	0.0699*	
H15	0.48395	0.08842	0.21743	0.0659*	
H16A	1.17937	−0.13571	0.21817	0.1318*	
H16B	1.18046	−0.22218	0.15981	0.1318*	
H16C	1.07976	−0.32495	0.20210	0.1318*	
H17A	0.82382	−0.19261	0.10015	0.1445*	
H17B	1.02683	−0.13118	0.09935	0.1445*	
H17C	0.89984	0.00571	0.11272	0.1445*	
H21	0.91542	0.32108	−0.00093	0.0922*	
H22	1.00947	0.38742	0.08848	0.0926*	
H23	0.80506	0.39089	0.15029	0.0818*	
H24A	0.29434	0.32966	0.00441	0.1161*	
H24B	0.26831	0.22737	0.05565	0.1161*	
H24C	0.31152	0.12950	0.00267	0.1161*	
H25A	0.69802	0.25302	−0.07345	0.1451*	
H25B	0.51451	0.31518	−0.06560	0.1451*	
H25C	0.53964	0.11889	−0.06001	0.1451*	
H26	0.58170	0.52033	0.17679	0.0697*	
H28	0.16784	0.25463	0.18562	0.0645*	
H29	−0.01497	0.27993	0.25174	0.0647*	
H31	0.34963	0.64904	0.33269	0.0716*	
H32	0.52481	0.62718	0.26472	0.0695*	
H33A	0.20414	0.54926	0.40369	0.1311*	
H33B	0.00866	0.56601	0.41478	0.1311*	
H33C	0.11592	0.71043	0.38523	0.1311*	
H34A	−0.19929	0.42302	0.29584	0.1281*	
H34B	−0.21486	0.46946	0.35692	0.1281*	
H34C	−0.15499	0.29064	0.33823	0.1281*	

### Atomic displacement parameters (Å^2^)

**Table:**

	*U*^11^	*U*^22^	*U*^33^	*U*^12^	*U*^13^	*U*^23^
N1	0.0518 (15)	0.0591 (16)	0.0505 (16)	0.0087 (12)	0.0085 (12)	0.0006 (12)
N2	0.0697 (18)	0.102 (2)	0.0511 (17)	0.0368 (16)	0.0067 (14)	0.0055 (15)
C1	0.0469 (18)	0.0497 (18)	0.059 (2)	0.0090 (14)	0.0074 (15)	0.0060 (15)
C2	0.0499 (18)	0.0507 (19)	0.0490 (19)	0.0084 (14)	0.0072 (15)	0.0090 (14)
C3	0.057 (2)	0.0542 (19)	0.055 (2)	0.0139 (15)	0.0119 (16)	0.0076 (15)
C4	0.054 (2)	0.076 (2)	0.073 (2)	0.0180 (17)	0.0192 (17)	0.0068 (18)
C5	0.0444 (19)	0.097 (3)	0.078 (3)	0.0096 (17)	0.0077 (18)	−0.002 (2)
C6	0.052 (2)	0.077 (2)	0.057 (2)	0.0054 (16)	0.0055 (16)	−0.0008 (16)
C7	0.0543 (19)	0.076 (2)	0.063 (2)	0.0072 (16)	0.0057 (15)	0.0011 (17)
C8	0.075 (2)	0.078 (2)	0.064 (2)	0.0201 (18)	0.0142 (17)	0.0019 (18)
C9	0.0502 (18)	0.0527 (19)	0.056 (2)	0.0058 (14)	0.0031 (15)	0.0012 (15)
C10	0.0477 (17)	0.0463 (18)	0.0480 (19)	0.0037 (14)	0.0033 (14)	0.0009 (14)
C11	0.0545 (18)	0.0499 (19)	0.0425 (17)	0.0011 (14)	−0.0002 (14)	−0.0007 (14)
C12	0.0510 (18)	0.0519 (19)	0.0510 (19)	0.0107 (14)	−0.0042 (15)	0.0016 (14)
C13	0.0515 (18)	0.0473 (18)	0.0507 (19)	0.0092 (14)	0.0017 (15)	−0.0019 (14)
C14	0.066 (2)	0.066 (2)	0.0430 (18)	0.0165 (17)	−0.0015 (16)	0.0047 (15)
C15	0.0554 (19)	0.057 (2)	0.054 (2)	0.0153 (15)	0.0008 (16)	0.0046 (15)
C16	0.074 (2)	0.117 (3)	0.083 (3)	0.038 (2)	0.0180 (19)	0.016 (2)
C17	0.111 (3)	0.137 (4)	0.055 (2)	0.060 (3)	0.018 (2)	0.008 (2)
N3	0.0573 (16)	0.0650 (18)	0.0542 (17)	0.0131 (13)	0.0075 (13)	0.0073 (14)
N4	0.0573 (17)	0.0783 (19)	0.0622 (18)	−0.0033 (14)	0.0069 (14)	−0.0101 (14)
C18	0.0513 (19)	0.058 (2)	0.060 (2)	0.0128 (15)	0.0114 (16)	0.0139 (15)
C19	0.059 (2)	0.059 (2)	0.060 (2)	0.0192 (15)	0.0086 (17)	0.0093 (16)
C20	0.077 (2)	0.071 (2)	0.064 (2)	0.0244 (19)	0.0170 (19)	0.0084 (17)
C21	0.069 (2)	0.086 (3)	0.085 (3)	0.022 (2)	0.033 (2)	0.016 (2)
C22	0.056 (2)	0.093 (3)	0.084 (3)	0.0107 (19)	0.012 (2)	0.008 (2)
C23	0.054 (2)	0.085 (3)	0.068 (2)	0.0129 (17)	0.0089 (18)	0.0069 (18)
C24	0.069 (2)	0.099 (3)	0.064 (2)	0.0188 (19)	0.0003 (17)	−0.0016 (19)
C25	0.106 (3)	0.126 (4)	0.067 (3)	0.035 (3)	0.023 (2)	0.011 (2)
C26	0.0519 (19)	0.059 (2)	0.065 (2)	0.0108 (16)	0.0071 (16)	0.0120 (17)
C27	0.0496 (18)	0.0497 (19)	0.057 (2)	0.0083 (14)	0.0018 (15)	0.0041 (15)
C28	0.0551 (19)	0.053 (2)	0.0500 (19)	0.0039 (15)	−0.0014 (15)	−0.0046 (14)
C29	0.0507 (18)	0.0520 (19)	0.055 (2)	−0.0017 (14)	−0.0007 (15)	−0.0032 (15)
C30	0.0458 (18)	0.0532 (19)	0.0522 (19)	0.0054 (14)	0.0025 (14)	0.0045 (15)
C31	0.056 (2)	0.061 (2)	0.055 (2)	−0.0044 (16)	−0.0022 (16)	−0.0118 (15)
C32	0.0452 (18)	0.058 (2)	0.067 (2)	−0.0029 (14)	0.0016 (16)	−0.0005 (17)
C33	0.092 (3)	0.095 (3)	0.068 (2)	−0.006 (2)	0.016 (2)	−0.026 (2)
C34	0.069 (2)	0.090 (3)	0.092 (3)	−0.010 (2)	0.0254 (19)	−0.010 (2)

### Geometric parameters (Å, °)

**Table:**

N1—C1	1.428 (4)	C16—H16B	0.9600
N1—C9	1.269 (4)	C16—H16C	0.9600
N2—C13	1.376 (4)	C17—H17B	0.9600
N2—C16	1.440 (4)	C17—H17C	0.9600
N2—C17	1.436 (4)	C17—H17A	0.9600
N3—C18	1.428 (4)	C18—C19	1.395 (4)
N3—C26	1.269 (4)	C18—C23	1.390 (4)
N4—C33	1.443 (4)	C19—C20	1.396 (5)
N4—C30	1.374 (4)	C19—C24	1.512 (4)
N4—C34	1.433 (4)	C20—C21	1.390 (5)
C1—C6	1.383 (4)	C20—C25	1.514 (5)
C1—C2	1.396 (4)	C21—C22	1.369 (5)
C2—C7	1.500 (4)	C22—C23	1.373 (5)
C2—C3	1.402 (4)	C26—C27	1.450 (4)
C3—C4	1.382 (4)	C27—C28	1.389 (4)
C3—C8	1.512 (4)	C27—C32	1.388 (4)
C4—C5	1.373 (5)	C28—C29	1.372 (4)
C5—C6	1.373 (4)	C29—C30	1.403 (4)
C9—C10	1.449 (4)	C30—C31	1.398 (4)
C10—C11	1.397 (4)	C31—C32	1.368 (4)
C10—C15	1.391 (4)	C21—H21	0.9300
C11—C12	1.367 (4)	C22—H22	0.9300
C12—C13	1.409 (4)	C23—H23	0.9300
C13—C14	1.396 (4)	C24—H24A	0.9600
C14—C15	1.367 (4)	C24—H24B	0.9600
C4—H4	0.9300	C24—H24C	0.9600
C5—H5	0.9300	C25—H25A	0.9600
C6—H6	0.9300	C25—H25B	0.9600
C7—H7B	0.9600	C25—H25C	0.9600
C7—H7A	0.9600	C26—H26	0.9300
C7—H7C	0.9600	C28—H28	0.9300
C8—H8A	0.9600	C29—H29	0.9300
C8—H8B	0.9600	C31—H31	0.9300
C8—H8C	0.9600	C32—H32	0.9300
C9—H9	0.9300	C33—H33A	0.9600
C11—H11	0.9300	C33—H33B	0.9600
C12—H12	0.9300	C33—H33C	0.9600
C14—H14	0.9300	C34—H34A	0.9600
C15—H15	0.9300	C34—H34B	0.9600
C16—H16A	0.9600	C34—H34C	0.9600
			
C1—N1—C9	120.0 (3)	H17A—C17—H17B	109.00
C13—N2—C16	121.6 (3)	N2—C17—H17A	109.00
C13—N2—C17	121.8 (3)	N2—C17—H17B	109.00
C16—N2—C17	116.6 (3)	N2—C17—H17C	109.00
C18—N3—C26	118.4 (3)	N3—C18—C19	117.8 (3)
C30—N4—C33	121.2 (3)	N3—C18—C23	121.5 (3)
C30—N4—C34	121.6 (3)	C19—C18—C23	120.7 (3)
C33—N4—C34	117.2 (3)	C18—C19—C20	118.9 (3)
C2—C1—C6	120.5 (3)	C18—C19—C24	120.3 (3)
N1—C1—C6	121.1 (3)	C20—C19—C24	120.8 (3)
N1—C1—C2	118.3 (3)	C19—C20—C21	119.1 (3)
C1—C2—C3	118.6 (2)	C19—C20—C25	120.9 (3)
C3—C2—C7	120.8 (2)	C21—C20—C25	120.0 (3)
C1—C2—C7	120.6 (2)	C20—C21—C22	121.7 (3)
C2—C3—C8	120.5 (3)	C21—C22—C23	119.6 (3)
C4—C3—C8	120.1 (3)	C18—C23—C22	120.1 (3)
C2—C3—C4	119.4 (3)	N3—C26—C27	124.1 (3)
C3—C4—C5	121.5 (3)	C26—C27—C28	123.7 (3)
C4—C5—C6	119.4 (3)	C26—C27—C32	119.6 (3)
C1—C6—C5	120.6 (3)	C28—C27—C32	116.7 (3)
N1—C9—C10	123.4 (3)	C27—C28—C29	121.8 (3)
C9—C10—C15	120.4 (2)	C28—C29—C30	121.0 (3)
C11—C10—C15	116.8 (2)	N4—C30—C29	121.6 (3)
C9—C10—C11	122.9 (3)	N4—C30—C31	121.2 (3)
C10—C11—C12	121.9 (3)	C29—C30—C31	117.2 (3)
C11—C12—C13	120.8 (3)	C30—C31—C32	120.7 (3)
C12—C13—C14	117.3 (3)	C27—C32—C31	122.5 (3)
N2—C13—C12	121.2 (3)	C20—C21—H21	119.00
N2—C13—C14	121.5 (3)	C22—C21—H21	119.00
C13—C14—C15	120.9 (3)	C21—C22—H22	120.00
C10—C15—C14	122.3 (3)	C23—C22—H22	120.00
C3—C4—H4	119.00	C18—C23—H23	120.00
C5—C4—H4	119.00	C22—C23—H23	120.00
C4—C5—H5	120.00	C19—C24—H24A	109.00
C6—C5—H5	120.00	C19—C24—H24B	109.00
C1—C6—H6	120.00	C19—C24—H24C	109.00
C5—C6—H6	120.00	H24A—C24—H24B	110.00
C2—C7—H7B	110.00	H24A—C24—H24C	109.00
C2—C7—H7C	109.00	H24B—C24—H24C	109.00
C2—C7—H7A	109.00	C20—C25—H25A	109.00
H7B—C7—H7C	109.00	C20—C25—H25B	109.00
H7A—C7—H7B	109.00	C20—C25—H25C	109.00
H7A—C7—H7C	109.00	H25A—C25—H25B	109.00
C3—C8—H8C	109.00	H25A—C25—H25C	109.00
H8B—C8—H8C	109.00	H25B—C25—H25C	109.00
H8A—C8—H8B	109.00	N3—C26—H26	118.00
H8A—C8—H8C	109.00	C27—C26—H26	118.00
C3—C8—H8B	109.00	C27—C28—H28	119.00
C3—C8—H8A	110.00	C29—C28—H28	119.00
N1—C9—H9	118.00	C28—C29—H29	120.00
C10—C9—H9	118.00	C30—C29—H29	120.00
C10—C11—H11	119.00	C30—C31—H31	120.00
C12—C11—H11	119.00	C32—C31—H31	120.00
C13—C12—H12	120.00	C27—C32—H32	119.00
C11—C12—H12	120.00	C31—C32—H32	119.00
C13—C14—H14	120.00	N4—C33—H33A	109.00
C15—C14—H14	120.00	N4—C33—H33B	109.00
C14—C15—H15	119.00	N4—C33—H33C	109.00
C10—C15—H15	119.00	H33A—C33—H33B	109.00
N2—C16—H16B	109.00	H33A—C33—H33C	110.00
N2—C16—H16C	109.00	H33B—C33—H33C	109.00
H16A—C16—H16B	109.00	N4—C34—H34A	109.00
H16A—C16—H16C	109.00	N4—C34—H34B	109.00
N2—C16—H16A	110.00	N4—C34—H34C	109.00
H16B—C16—H16C	109.00	H34A—C34—H34B	110.00
H17A—C17—H17C	109.00	H34A—C34—H34C	109.00
H17B—C17—H17C	109.00	H34B—C34—H34C	109.00
			
C9—N1—C1—C2	−145.2 (3)	C9—C10—C11—C12	−177.9 (3)
C9—N1—C1—C6	39.0 (4)	C10—C11—C12—C13	0.4 (4)
C1—N1—C9—C10	−176.4 (3)	C11—C12—C13—N2	176.6 (3)
C16—N2—C13—C14	175.9 (3)	C11—C12—C13—C14	−2.0 (4)
C16—N2—C13—C12	−2.6 (5)	N2—C13—C14—C15	−176.8 (3)
C17—N2—C13—C12	178.6 (3)	C12—C13—C14—C15	1.8 (5)
C17—N2—C13—C14	−2.9 (5)	C13—C14—C15—C10	0.0 (5)
C26—N3—C18—C23	38.3 (4)	N3—C18—C19—C24	2.5 (4)
C18—N3—C26—C27	−176.9 (3)	C23—C18—C19—C20	−1.7 (5)
C26—N3—C18—C19	−145.2 (3)	N3—C18—C19—C20	−178.3 (3)
C34—N4—C30—C31	169.5 (3)	N3—C18—C23—C22	178.2 (3)
C33—N4—C30—C31	−14.2 (4)	C19—C18—C23—C22	1.8 (5)
C33—N4—C30—C29	166.9 (3)	C23—C18—C19—C24	179.0 (3)
C34—N4—C30—C29	−9.5 (4)	C24—C19—C20—C21	179.7 (3)
C2—C1—C6—C5	−1.2 (5)	C18—C19—C20—C21	0.4 (5)
C6—C1—C2—C7	179.3 (3)	C18—C19—C20—C25	−177.7 (3)
N1—C1—C6—C5	174.4 (3)	C24—C19—C20—C25	1.5 (5)
N1—C1—C2—C3	−175.2 (3)	C25—C20—C21—C22	179.1 (3)
C6—C1—C2—C3	0.6 (4)	C19—C20—C21—C22	0.9 (5)
N1—C1—C2—C7	3.5 (4)	C20—C21—C22—C23	−0.9 (5)
C7—C2—C3—C4	−178.8 (3)	C21—C22—C23—C18	−0.4 (5)
C7—C2—C3—C8	−0.4 (4)	N3—C26—C27—C28	−7.5 (5)
C1—C2—C3—C4	−0.2 (4)	N3—C26—C27—C32	172.2 (3)
C1—C2—C3—C8	178.3 (3)	C26—C27—C28—C29	−177.5 (3)
C8—C3—C4—C5	−178.2 (3)	C32—C27—C28—C29	2.8 (5)
C2—C3—C4—C5	0.3 (5)	C26—C27—C32—C31	178.7 (3)
C3—C4—C5—C6	−0.9 (5)	C28—C27—C32—C31	−1.6 (5)
C4—C5—C6—C1	1.3 (5)	C27—C28—C29—C30	−1.1 (5)
N1—C9—C10—C11	1.8 (4)	C28—C29—C30—N4	177.2 (3)
N1—C9—C10—C15	−177.5 (3)	C28—C29—C30—C31	−1.7 (4)
C9—C10—C15—C14	177.8 (3)	N4—C30—C31—C32	−176.1 (3)
C11—C10—C15—C14	−1.6 (4)	C29—C30—C31—C32	2.9 (4)
C15—C10—C11—C12	1.4 (4)	C30—C31—C32—C27	−1.2 (5)

### Hydrogen-bond geometry (Å, °)

**Table:**

Cg1, Cg2 and Cg4 are the centroids of the C1–C6, C10–C15 and C27–C32 benzene rings, respectively.

**Table:**

*D*—H···*A*	*D*—H	H···*A*	*D*···*A*	*D*—H···*A*
C7—H7A···Cg1^i^	0.96	2.90	3.756 (3)	149
C16—H16C···Cg4^ii^	0.96	2.69	3.434 (4)	135
C32—H32···Cg2^iii^	0.93	2.88	3.698 (3)	148

Symmetry codes: (i) −*x*+1, −*y*, −*z*+1; (ii) *x*+1, *y*−1, *z*; (iii) *x*, *y*+1, *z*.
